# Contemporary use of prophylactic probiotics in NICUs in the United States: a survey update

**DOI:** 10.1038/s41372-024-01952-0

**Published:** 2024-03-29

**Authors:** Samantha J. Wala, Mecklin V. Ragan, Erin Pryor, Jennifer Canvasser, Karen A. Diefenbach, Gail E. Besner

**Affiliations:** 1 Center for Perinatal Research, Nationwide Children’s Hospital, Columbus, OH USA; 2 Department of Pediatric Surgery, Nationwide Children’s Hospital, Columbus, OH USA; 3 NEC Society, Davis, CA USA

**Keywords:** Paediatrics, Nutritional supplements

## Abstract

**Objective:**

In 2015, 14.0% of US NICUs administered probiotics to very low birth weight infants. Current probiotic use prior to and after the Fall of 2023 (when FDA warnings were issued) remains unknown.

**Study design:**

A survey was distributed to the American Academy of Pediatrics Section on Neonatal and Perinatal Medicine (August–November/2022) and Neonatology Solutions’ Level III/IV NICUs (January–April/2023). Probiotic administration practices were investigated.

**Results:**

In total, 289 unique NICUs and 406 providers responded to the survey. Of those, 29.1% of NICUs administered prophylactic probiotics to premature neonates, however, this decreased considerably after FDA warnings were issued. Additionally, 71.4% of providers stated willingness to administer probiotics to premature infants if there was an FDA-approved formulation.

**Conclusions:**

Probiotic use in US NICUs increased between 2015 and the Fall of 2023 and then dropped dramatically following warning letters from the FDA. The introduction of an FDA-approved probiotic may further expand administration.

## Introduction

Intestinal colonization in a neonate is a dynamic process that has important consequences on infant health. Preterm neonates have reduced microbial diversity [1]. In addition, the preterm intestine is predisposed to colonization by pathogenic facultative anaerobes, such as *Enterobacter*, *Escherichia*, and *Klebsiella*, and underrepresentation of the early colonizing, strictly anaerobic commensal organisms, including *Bifidobacterium, Bacteroides*, and *Clostridium* [2, 3]. The latter species are important in educating the developing immune system and facilitating colonization by subsequent microbes [4]. Factors associated with prematurity, such as cesarean delivery, antibiotic exposure, parenteral nutrition, and the use of formula feeds, have been linked to dysbiosis in premature infants [5]. Breast milk has been associated with expansion of beneficial lactic acid producing bacteria, such as *Lactobacillus* [6]. Furthermore, neonatal dysbiosis has been associated with certain disease processes, such as necrotizing enterocolitis (NEC), late-onset sepsis, feeding intolerance, and neurodevelopmental impairment [7–10].

Given the dysbiosis present in premature neonates, probiotics have a significant impact on their gut microbiome. The Food and Agriculture Organization of the United Nations and the World Health Organization (WHO) define probiotics as “live microorganisms that, when administered in adequate amounts, confer a health benefit on the host” [11]. Probiotics appear to have positive effects, including reducing intestinal inflammation, promoting gut microbiome maturation and stability, inhibiting colonization by pathogenic bacteria, and regulating the innate immune response [12–14]. In addition, the use of probiotics appears to be effective given that their administration has been shown to readily modify the early-life microbiota in preterm infants [15, 16]. Therefore, probiotics may have a significant beneficial impact on the health of premature infants.

For over two decades, prophylactic probiotic administration has been investigated for use in the premature patient population. Multiple randomized controlled trials (RCTs) have studied the ability of probiotics to prevent late-onset sepsis and NEC [17–21]. A meta-analysis did demonstrate that probiotics may reduce the risk for NEC in very preterm or very low birth weight (VLBW) infants, although with low certainty of evidence [22]. Nonetheless, the results are conflicting. This is most likely due to variability in probiotic products and administration practices between RCTs. Furthermore, there are currently no Food and Drug Administration (FDA)-approved, pharmaceutical-grade, Good Manufacturing Practice (GMP)-grade probiotics approved for use. In November 2021, the American Academy of Pediatrics (AAP) published the following on the use of probiotics in preterm infants: “Given the lack of FDA-regulated pharmaceutical-grade products in the United States, conflicting data on safety and efficacy, and potential for harm in a highly vulnerable population, current evidence does not support the routine, universal administration of probiotics to preterm infants, particularly those with a birth weight of <1000 g” [23]. On the other hand, the European Society for Pediatric Gastroenterology Hepatology and Nutrition published a position paper supporting the conditional use of probiotics to prevent NEC, provided that quality is assured and safety issues are met [24]. Similarly, the American Gastroenterological Association recommends conditional use of *Lactobacillus* spp and *Bifidobacterium* spp in preterm infants for the prevention of NEC [25]. The WHO also recently published a new recommendation that probiotics formulated for preterm or low birthweight infants be considered for use in infants <32 weeks gestation who are fed human milk [26]. Despite the recent AAP statement, probiotics continue to be used in NICUs across the US. According to the Vermont Oxford Network database, in 2013, 5.2% of NICUs were using probiotics [27]. This increased to 6.7% in 2014. Between May and September 2015, Viswanathan et al. [27] conducted a phone survey and found that 14.0% (70/500) of NICUs in the US used probiotics in VLBW infants. Furthermore, they showed that 90% of the probiotics used in the NICUs were not studied in RCTs that recruited VLBW infants. To assess the impact of the AAP statement on probiotic administration and current patterns of probiotic use, Hanna et al. [28] surveyed 430 NICUs that were a part of the Children’s Hospital Neonatal Consortium (CHNC) or Pediatrix Medical Group between February and October 2022, with responses from 95 NICUs. While only a specific subset of NICUs was surveyed, resulting in a small sample size, 39% of NICUs reported currently using probiotics, and 24% indicated that the AAP statement “significantly influenced their decision regarding probiotic use.” Interestingly, 37% of NICUs not administering probiotics currently were considering initiating probiotic administration protocols [28].

In the current study, we provide a broader picture of the number of NICUs administering prophylactic probiotics to premature infants. We also characterize different practices of probiotic administration, and providers’ opinions regarding use of an FDA-approved probiotic if one were to exist.

## Materials and methods

This study was approved by the Institutional Review Board (STUDY00002627) at Nationwide Children’s Hospital, Columbus, Ohio. The study was performed in accordance with the Declaration of Helsinki. To identify NICUs administering prophylactic probiotics, a cross-sectional REDCap survey (Vanderbilt University, Nashville, TN and National Institutes of Health, Bethesda, MD) was first distributed to all members of the AAP Section on Neonatal and Perinatal Medicine (SONPM) between August and November 2022. As per AAP guidelines, the survey was distributed by the AAP SONPM Section Manager. It was sent to all 4532 members of the AAP SONPM, the vast majority of whom are neonatologists and maternal-fetal medicine (MFM) specialists in practice and in training. We were unable to discern the number of neonatologists *vs*. MFM specialists that received the survey, as this information was not available to us. In addition, we emailed the survey to NICU representatives of 580 Level III and Level IV NICUs from January to April 2023, with accessible contact information identified by the Neonatology Solutions NICU Directory (https://neonatologysolutions.com/nicu-directory/; accessed January 2023). The point of contact for the survey was either the attending neonatologist, neonatology fellow, NICU medical director, neonatal nurse practitioner, NICU nurse manager, or dietitian.

Data were collected regarding specific probiotic administration protocols, including dosing and frequency, the commercial product being used, and adverse events. Individual provider opinions regarding the influence of the AAP statement on probiotic use, and whether an FDA-approved probiotic option would affect provider use were also gathered. Inclusion of the name, affiliated institution, and email address was optional for survey respondents. A question regarding the gestational age at which prophylactic probiotics were initiated was included after distribution of the survey to providers who had entered an email address. In addition, due to the recent FDA warnings regarding probiotics issued in September - October 2023, we sent a follow-up survey to this subset of respondents in December 2023 to determine whether they were still providing probiotics in their NICUs. All individual provider responses were included in provider specific analysis. For institutional analysis, only the most recent response for each institution was used.

## Results

A total of 406 providers responded to the survey, representing 289 unique institutions. Ninety point nine percent (90.9%; 369/406) of respondents were attending neonatologists, 5.9% (24/406) were NICU fellows, and 3.2% (13/406) were other (NICU medical director, neonatal nurse practitioner, NICU nurse manager, dietitian). Overall, 59.9% (173/289) of respondent’s institutions were Level III NICUs and 40.1% (116/289) were Level IV NICUs. Twenty-nine point one percent (29.1%; 84/289) of responding institutions reported administering prophylactic probiotics to premature infants less than 37 weeks gestational age (Table 1). Of those 84 NICUs, 98.8% (83/84) reported administering prophylactic probiotics using a standardized protocol (Table 1). From those 83 NICUs with a standardized protocol, 27.7% (23/83) administered probiotics with human milk only, whereas 71.1% (59/83) provided probiotics with human milk or formula (Table 1). Almost all NICUs administered probiotics using a non-weight-based dose (92.8%, 77/83) (Table 1). Probiotics were dosed once daily with feeds in 85.5% (71/83) of institutions, once daily not with feeds in 7.2% (6/83) of institutions, and multiple times daily with feeds in 7.2% (6/83) of institutions (Table 1). Factors that affected probiotic dose or frequency included birth weight, current weight, health status, and feeding status, such as whether the neonate is nil per os or being fed enterally or parenterally.

**Table 1:** Characterization of prophylactic probiotic use in US NICUs.

Survey questions	*n* (%)
Administration of prophylactic probiotics	*n* = 289
Yes	84 (29.1%)
No	205 (70.9%)
NICUs with a standardized probiotic administration protocol (*n* = 84)	83 (98.8%)
Probiotics administered with feeds (*n* = 83)	
With human milk only	23 (27.7%)
With human milk or formula	59 (71.1%)
Other	14 (16.9%)
Probiotic dosing (*n* = 83)	
Weight-based	5 (6.0%)
Non-weight based	77 (92.8%)
Other	2 (2.4%)
Probiotic administration frequency (*n* = 83)	
Once daily, not with feeding	6 (7.2%)
Once daily, with feeding	71 (85.5%)
Multiple times daily, with feeding	6 (7.2%)
Reason for administration (*n* = 84)	
Reduce the risk of NEC	83 (98.8%)
Reduce the risk of sepsis	34 (40.5%)
Decrease feeding intolerance	37 (44.0%)
Other	4 (4.8%)

The four most reported probiotics administered were: (1) Similac^®^ Probiotic Tri-Blend consisting of *Bifidobacterium lactis* (*B. lactis*)*, B*. *infantis*, and *Streptococcus thermophilus* (30.4%, 24/79), (2) Evivo^®^ consisting of *B*. *infantis* (13.9%, 11/79), (3) Ultimate Flora^TM^ (formerly, FloraBaby) consisting of *B*. *breve*, *Lactobacillus rhamnosus* (*L*. *rhamnosus*), *B*. *bifidum, B*. *infantis*, and *B*. *longum* (12.7%, 10/79), and (4) FloraTummys^®^ consisting of *B*. *lactis and L*. *acidophilus* (8.9%, 7/79). Almost all NICUs reported administering probiotics to reduce the risk of NEC (98.8%, 83/84) (Table 1). Other reasons for prophylactic probiotic administration were to decrease feeding intolerance (44.0%, 37/84) and to reduce the risk of sepsis (40.5%, 34/84). The gestational age at which probiotics were administered varied widely, with 9.8% (4/41) administering probiotics to all gestational ages, 31.7% (13/41) to <32 weeks, 7.3% (3/41) to <33 weeks, 31.7% (13/41) to <34 weeks, and 9.8% (4/41) to <35 weeks. There were five adverse events consisting of sepsis of unknown origin. Based on the information provided, it is unclear whether the sepsis that occurred in all of these cases was due to the actual probiotic administered, however, there was one death of an extremely low birth weight (ELBW) infant from sepsis secondary to *B*. *infantis* contained in the probiotic formulation.

Forty-five percent (184/406) of respondents indicated that their stance on probiotic administration was impacted by the AAP’s statement on probiotics (Table 2). Most providers indicated that FDA-approval of a probiotic formulation would be very important (52.0%, 211/406) or somewhat important (32.8%, 133/406) (Table 2). In addition, 71.4% (290/406) of providers indicated that FDA approval of a probiotic preparation would influence their willingness to routinely administer probiotics to premature infants. Providers were specifically asked about their willingness to administer a new FDA-approved probiotic for NEC if it was predicted to decrease the incidence of NEC by different percentages. Most providers indicated they would be willing to administer an FDA-approved probiotic if it decreased the incidence of NEC by as little as 10% (25.1%, 102/406) or 25% (31.8%, 129/406) (Table 2). Importantly, after the recent FDA warnings, only 2 out of 60 providers that received a secondary survey confirmed that they are still providing probiotics (one using Similac^®^ Probiotic Tri-Blend and one using FloraTummys^®^).

**Table 2:** Characterization of opinions towards AAP probiotic statement and an FDA-approved probiotic formulation.

Survey questions	*n* (%)
Impact of AAP probiotics statement on provider stance on probiotic use	*n* = 406
Yes	184 (45.3%)
No	222 (54.7%)
Importance of an FDA-approved probiotic formulation for administration	
Very important	211 (52.0%)
Somewhat important	133 (32.8%)
Neutral	50 (12.3%)
Not important	12 (3.0%)
Influence of an FDA-approved probiotic formulation for administration	
Yes	290 (71.4%)
No	35 (8.6%)
Already routinely administer probiotics	81 (20.0%)
Willingness to institute a new FDA-approved probiotic if it decreased the incidence of NEC by as little as:	
10%	102 (25.1%)
25%	129 (31.8%)
50%	62 (15.3%)
75%	8 (2.0%)
>75%	31 (7.6%)
Already routinely administer probiotics	74 (18.2%)

## Discussion

In 2015, it was reported that 14.0% of NICUs administered prophylactic probiotics to VLBW infants in NICUs [27]. A study earlier this year showed that 39% of 95 responding NICUs that were part of CHNC or Pediatrix Medical Group administered probiotics to VLBW infants [28]. We now report that 29.1% of 289 responding NICUs were administering prophylactic probiotics to premature neonates prior to the Fall of 2023. Although it is challenging to make a direct comparison between these different surveys, there does appear to be an increase in prophylactic probiotic use in NICUs since 2015. Since the publication of the 2015 survey, there have been additional RCTs evaluating the benefits of prophylactic probiotic use in premature neonates [29, 30]. Additionally, our knowledge about probiotics has expanded over the last few years [31]. In comparison to the previous survey, there is also an increase in the use of multi-strain compared to single strain probiotics. This is unsurprising given that there have been compelling results with the use of multi-strain probiotic formulations [32–34].

The survey conducted by Viswanathan et al. [27] revealed that only four out of the sixteen probiotics identified as being administered to VLBW infants in NICUs had been evaluated in a RCT. Similar to Hanna et al. [28], we found that Similac^®^ Probiotic Tri-Blend, composed of *B*. *infantis*, *Streptococcus thermophilus*, and *B*. *lactis*, was the most used probiotic for prophylaxis amongst our respondent institutions. Although this commercial product has not been studied in an RCT in the US, this specific probiotic combination produced by Solgar^®^ (Leonia, NJ) has been evaluated in the multi-center, randomized, double-blinded, placebo-controlled ProPrems trial in Australia and New Zealand [18]. In that study, there was no significant change in late-onset sepsis, but there was a significant decline in the incidence of NEC (Bell stage ≥2) in the probiotic group compared to control. The second most used probiotic in our study was Evivo^®^, which contains *B. longum* subspecies *infantis* EVC001. There have been studies that encourage the use of this probiotic to help prevent NEC [35, 36]. The IMPRINT study was a phase 1 clinical trial that demonstrated the safety and tolerability of *B. longum* subspecies *infantis* EVC001 administration for 21 consecutive days in healthy, full-term infants [37]. The third most common was Ultimate Flora^TM^ (formerly, FloraBABY), which consists of *B*. *breve*, *L*. *rhamnosus*, *B*. *bifidum*, *B*. *infantis*, and *B*. *longum*. This commercial product was also one of the most administered probiotics in 2015 [27]. An RCT showed that FloraBABY can accelerate maturation of the microbiome in extremely premature infants to a state that more closely resembles vaginally born, breastfed infants [12]. Moreover, studies have shown that this probiotic formulation can significantly decrease the incidence of NEC in premature infants [38, 39]. The fourth most common probiotic was FloraTummys^®^, which contains *L*. *acidophilus* and *B*. *infantis*. In addition, this combination of bacteria has been studied in a prospective RCT using Infloran^®^ (Swiss Serum and Vaccine Institute, Berne, Switzerland) in Taiwan [17]. In 367 VLBW infants, the incidence of death, NEC, and sepsis were significantly lower in the probiotic group compared to the control group. Therefore, we found that although the specific bacteria within the most commonly used commercial probiotic products have been studied in clinical trials, the specific commercial products have not been directly studied in clinical trials.

Regarding probiotic practices, we found that only one responding NICU that did administer probiotics did so without a standardized protocol for probiotic administration. Nonetheless, there was variability in practice between different NICUs in terms of administering probiotics with human milk or formula, frequency of dosing, the commercial product being used, and the gestational age at which prophylactic probiotics were initiated. These variances in practice highlight an opportunity to standardize prophylactic probiotic usage among NICUs across the US.

Although the number of NICUs across the US that were currently administering probiotics prophylactically to premature infants increased between 2015 to Fall 2023, it was still small. In the current survey, the majority of respondents indicated that FDA approval of a probiotic preparation would influence their willingness to routinely administer probiotics to premature infants. In 2016, Lewis et al. conducted a study to validate the identity of *Bifidobacterium* species and subspecies in 16 commercial probiotic products, and found that only 1 of the 16 products perfectly matched its label [40]. This further demonstrates the importance of having an FDA-approved probiotic formulation. One of the most significant concerns regarding probiotic use is related to the risk of sepsis. There have been reports of infant mortality due to sepsis secondary to infection caused by either the probiotic delivered or a fungal contaminant contained in the probiotic preparation administered [41–44]. In addition, after our initial survey was completed, warnings from the FDA were released regarding the use of probiotics in preterm infants due to the risk that microorganisms contained in probiotics can cause bacteremia or fungemia. An FDA warning issued on September 29, 2023 to healthcare providers highlighted the recent death of a premature baby receiving Evivo^®^ with medium-chain triglyceride (MCT) oil due to sepsis caused by *B*. *longum* subspecies *infantis*, which was a genetic match to the bacteria contained in the probiotic preparation administered (https://www.fda.gov/safety/medical-product-safety-information/risk-invasive-disease-preterm-infants-given-probiotics-formulated-contain-live-bacteria-or-yeast; accessed October 2023). An FDA warning letter to Infinant Health issued on September 28, 2023 resulted in the voluntary recall of Evivo^®^ with MCT oil from the market. Further, an FDA warning to Abbott Laboratories on October 24, 2023 led to discontinued sales of Similac^®^ Probiotic Tri-Blend. Thus, in the U.S., the two most commonly used probiotic preparations are no longer available for use; however, they remain available outside of the U.S. These warnings would have certainly changed the results of our initial survey had our survey been performed *after* the release of these warnings. To better characterize the effects of the FDA warnings, a subset of our initial respondents was surveyed, and only two of 60 respondents re-surveyed were still providing probiotics to premature infants.

The recent FDA warnings state that “In the absence of an approved product, healthcare providers who administer products containing live bacteria or yeast to treat, mitigate, cure or prevent a disease or condition are required to submit an Investigational New Drug application to the agency to ensure the investigational use of an unapproved product is conducted with the appropriate safeguards.” There remains an urgent need to reduce the risk of NEC in preterm infants, and an FDA-approved probiotic formulation for use in this patient population will be beneficial. The FDA should work with manufacturers to ensure that this occurs. The estimated cost for research and development of an FDA-approved product between 2009 and 2018 was $985 million [45]. Moreover, any therapy for premature infants must be studied in a phase 1 clinical trial in adults prior to a phase 1 clinical trial in newborns, thereby significantly increasing the cost for drug development in the newborn patient population [6]. Thus, probiotic manufacturers are currently not motivated to produce a probiotic that meets the definition of a drug due to the extremely high cost and effort involved, and because their primary market is healthy outpatients rather than preterm infants. However, given the multiple and diverse reported benefits of probiotic administration on the gut and on neurological development in premature babies at risk of NEC, companies should be encouraged to invest in an FDA-approved probiotic formulation, since from a provider standpoint the need is extraordinary. Most individual respondents indicated that they would be willing to administer an FDA-approved probiotic even if it only reduced the incidence of NEC by as little as 10–25%. This response highlights the severity of NEC, and the desperation of caregivers to find a solution for patients and their families.

There are additional limitations to the data presented in this study. The findings shown represent results from providers who replied to the survey only. We limited the survey to Level III and Level IV NICUs because they are most likely to care for premature neonates with NEC, the main reason that respondents cited for administering prophylactic probiotics. Data on the gestational age at which prophylactic probiotics are initiated were limited to a subgroup of respondents who provided their name and/or email address from institutions that administer prophylactic probiotics. Moreover, this is not an exhaustive survey, and other variations in practice between NICUs with respect to probiotic administration most likely exist. In addition, given the recent FDA warnings on probiotics, the use of probiotics in the U.S. has nearly halted.

In conclusion, we found that 29.1% of Level III and Level IV NICUs surveyed provided routine prophylactic probiotics to premature neonates at the time that our initial survey was conducted. However, recent FDA warnings regarding probiotic use in premature infants has nearly eliminated the use of this potentially life-saving therapy in the U.S. Improved prevention strategies, including implementation of FDA-approved probiotic formulations, are therefore crucial. The wide-ranging reported benefits of probiotic administration provides an incentive for increased investment in the research and development of safe and effective probiotics for preterm infants.

### Parent’s perspective

Patient-families with children diagnosed with NEC intimately understand this disease’s devastating, life-altering effects and recognize the urgent need for improved prevention options. Given the menacing nature of NEC and the current science, the NEC Society urges NICUs, professional associations, and clinicians to integrate and engage families in decisions that have life-altering implications, including the use of probiotics. The NEC Society (NECsociety.org) is a trailblazing patient-led nonprofit organization dedicated to accelerating NEC research, education, and advocacy. It is essential for multidisciplinary stakeholders to work together to improve prevention strategies and optimize NEC outcomes. Parents are the most important member of their child’s NICU team, and open communication with families is vital when considering acceptable risks and benefits of various treatment options. This survey asked respondents whether a deliberate effort was made to ensure that families are informed about the NICU’s probiotic procedures. Sixty-five point five percent (65.5%; 55/84) of institutions replied YES, 20.2% (17/84) only provided information if families asked, and 13.1% (11/84) answered NO.

There is a growing awareness of probiotics due to marketing, media coverage, and research. Accordingly, families of VLBW infants should be better informed about probiotics’ potential to help prevent NEC and death. The NEC Society’s Neonatal Probiotics Toolkit (https://necsociety.org/neonatal-probiotics-toolkit; accessed October 2023) was designed to support clinicians and units through the complex decision of probiotic use, and how to effectively communicate with families. However, given the FDA’s recent warnings on probiotic use in preterm infants, families and clinicians find themselves with one less tool to prevent NEC. Given the paucity of promising medications and treatments for vulnerable neonates at risk of NEC, physicians working in partnership with their patient-families should decide how currently available treatments or tools, such as dietary supplements, drugs, and other therapies, are used for complex patients in the NICU, considering the best available evidence. Pursuing safety is essential and must be balanced with the realization that fixation on perfection leaves preterm infants less protected from the devastation of NEC. It is imperative for governmental organizations and the broader neonatal community to acknowledge and appropriately respond to the overwhelming, tragic burden of NEC. Despite obstacles and setbacks, we urge the field not to waver in our shared goal of preventing NEC and improving outcomes for all infants in the NICU.

## Data Availability

The datasets generated during and/or analysed during the current study are not publicly available due to privacy concerns for survey respondents and their representative institutions but are available from the corresponding author on reasonable request.
